# From Expansion to Expiration: Federal Policy Waivers and Telehealth Utilization in US Primary Care (2016–2024)

**DOI:** 10.63116/RCTG8117

**Published:** 2026-02-12

**Authors:** Abhilasha Bellapu, John Geracitano, Jamie Conklin, Saif Khairat

**Affiliations:** 1 Gillings School of Global Public Health, University of North Carolina at Chapel Hill, Chapel Hill, NC, USA; 2 Carolina Health Informatics Program, University of North Carolina at Chapel Hill, Chapel Hill, NC, USA; 3 Health Sciences Library, University of North Carolina at Chapel Hill, Chapel Hill, NC, USA; 4 School of Nursing, University of North Carolina at Chapel Hill, Chapel Hill, NC, USA; 5 Sheps Center for Health Services Research, University of North Carolina at Chapel Hill, Chapel Hill, NC, USA

**Keywords:** telehealth, policy waivers, Medicare, Medicaid, synchronous telemedicine, primary care, health equity

## Abstract

**Background:**

The use of Telehealth has fluctuated over the past 8 years, with events such as the COVID-19 pandemic driving considerable growth in usage. During the COVID-19 pandemic, federal policy waivers were enacted to have Medicare cover costs for synchronous telehealth visits, increasing accessibility to telehealth during a public health emergency. Although waivers temporarily expanded access, sustainable, equitable telehealth access continues to heavily depend on overlooked factors such as technological accessibility, broadband availability, disability accommodations, and insurance status, particularly in rural and underserved populations. With these federal waivers set to expire on December 31, 2025, it is critical to evaluate the effectiveness and long-term impact of such federal policy waivers and to determine whether these have meaningfully improved telehealth access or have merely delayed underlying inequities.

**Methods:**

In this scoping review, the authors aimed to evaluate the impact of federal policy waivers on the utilization of synchronous telehealth with a focus on primary care across the United States from 2016 to 2024. Database searches across PubMed, SCOPUS, Cumulative Index of Nursing and Allied Health Literature (CINAHL), and PsycINFO yielded 137 unique references. Two screening stages resulted in 19 articles that met all evaluation criteria for review inclusion.

**Results:**

A review of the existing literature showed a common theme, in that federal waivers primarily affected telehealth usage by altering key social determinants of health and structural accessibility—driving both overuse in some populations and underuse in others. The most significant impact of the waivers was removal of geographic restrictions, particularly benefiting disadvantaged and rural communities. Studies noted that following the expiration of the waivers, patients—especially those seeking care across state lines—were less likely to attend either telemedicine or in-person visits, highlighting an immediate reduction in care access.

**Conclusions:**

Overall, previous studies have emphasized that sustaining telehealth access depends less on awareness efforts and more on addressing implementation barriers, particularly for nonambulatory, transportation-dependent, and financially disadvantaged populations.

## BACKGROUND

Telehealth has leveled the playing field in creating an accessible alternative to traditional in-person appointments, meeting patients right where they are while still providing quality care.^1^ On paper, the impact of telehealth is undeniable, with beneficiaries of Medicare who used telehealth care growing from a startling 13 000 to approximately 1.7 million by May 2020, just 2 months into a federal policy waiver enacted during the COVID-19 pandemic to have costs covered for synchronous telehealth visits, increasing accessibility to telehealth during a public health emergency (PHE).^1^ What was considered a “small proportion of care” has now become a necessity in the medical space, with over 87% of hospitals reporting telehealth as an active, repeatedly used tool.^2^ The federal policy waivers that enabled vulnerable populations to receive timely care and largely influenced the rise in telehealth use^2^ are effective through December 2025, but the effects of these waivers on telehealth utilization and its implications to guide future policymakers, providers, and researchers have not yet been completely examined. Thus, the true, full picture of how equitable telehealth access is for the patients behind these increased numbers is still not entirely clear.

Despite widespread adoption of telehealth, the incentives for its continued expansion have become less apparent with the COVID-19 pandemic no longer classified as a national PHE.^3^ Notably, if Congress no longer sees the need for telehealth and fails to extend these waivers past its expiration date of December 31, 2025, a “telehealth cliff” could result, leading to a significant reduction in access, benefits, and coverage for telehealth services across the country.^4^

In 2020, the 1135 telehealth policy waivers, the Coronavirus Preparedness and Response Supplemental Appropriations Act, and Section 1812 of the Social Security Act increased Medicare beneficiaries’ access to telehealth by waiving key geographic, monetary, and eligibility restrictions.^2^ In March 2020 alone, Medicare seemed to have become more lenient with its conditions, in that it expanded telehealth coverage and added a number of additional services to its expanded coverage and services list.^5^ Before the waivers, Medicare, Medicaid, and private carriers reimbursed telemedicine costs across the United States, yet the scope of what the coverages entailed was limited and disparate.^1,6^ Not all patients could receive the same benefits or coverage despite the so-called reimbursements. Yet, under the federal policy waivers, new patients who did not already have an established relationship with providers or patients who hoped to receive services outside of their state of enrollment were all accepted.^1,6^ Under these changes, in-person visits were almost fully replaced by the virtual options.^2^ These waivers allowed providers to triage more serious ailments that required their physical presence and enabled more patients to be treated in time and with efficiency.

With the popularity of telehealth exponentially growing, its nuances, technological advances, and definitions have accordingly expanded.^2^ Although the focus has been on the broader category of telehealth, the subcategory of telemedicine is also considered throughout this scoping literature review, as it is a key component of telehealth and represents a significant portion of its usage.

Moreover, with the exponential use of telehealth, factors that either prevent or encourage patients to use this facility have also increased. Several key variables affect telehealth adoption.

Examples include insurance coverage and status, geographic location, and awareness of services.^7^ For instance, dual eligibility status, which means a patient has eligibility for both Medicare and Medicaid benefits, could be the reason patients are more likely to have had video telehealth encounters.^8^ Similarly, a patient’s geographic status, especially if they were living in nonmetropolitan areas, indicates that patients are less likely to receive the option of using telehealth.^2^ Lack of awareness is another barrier, with 75% of consumers in a national survey on telemedicine utilization thinking their insurance did not offer or cover telemedicine and 35% unaware of the services even being offered—a disparity more apparent in rural and suburban areas compared with urban areas.^1^

Quantitative measures such as the Area Deprivation Index (ADI) help identify the comprehensive socioeconomic factors and disparities—factoring in education, employment, household income, and housing quality to present a holistic picture rather than relying on individual markers.^6^ A higher ADI score indicates a higher level of deprivation, socioeconomic barriers, and disadvantage, whereas a lower ADI score suggests affluence and less socioeconomic disadvantages.^6^ Measures such as these allow individual statistics to become better contextualized and better studied.

These evident disparities raise a more critical question of just how equitable access to telehealth is versus disproportional access to telehealth, in certain populations. This scoping review aims to identify the factors influencing telehealth utilization in primary care, highlighting the interplay between access, equity, and policy to inform future discussion and decisions on telehealth utilization. Because of the increase in the types of telehealth applications, we focused on synchronous telehealth and telemedicine, with real-time communication occurring between the patient and their provider.^9^

## METHODS

This scoping review was designed to assess the impact of federal policy waivers on synchronous telehealth utilization in US primary care from 2016 to 2024 and to evaluate the equity of telehealth services and access during this period.

### Databases

In October 2024, PubMed, Scopus, Cumulative Index of Nursing and Allied Health Literature (CINAHL), and PsycINFO were searched to identify relevant literature regarding telehealth utilization patterns and factors from 2016 to 2024 (Appendix A).

### Eligibility Criteria

Studies published before 2016, studies missing full text during the review period, and studies published in languages other than English were excluded. In addition, studies that discussed telehealth outside of a primary care or general context were excluded. Studies that provided a background of telehealth policy waivers and discussed the implications of the waivers at present, before the COVID-19 pandemic, and specifically after the COVID-19 pandemic were included. Furthermore, studies that discussed the direct and/or indirect factors that affected telehealth utilization, such as sex, demography, geography, were included.

### Search Strategy

Key search terms that correspond to the research question were (1) telemedicine, (2) waivers, (3) digital interventions, (4) mobile health, (5) mobile health applications, (6) teleconferencing, (7) teleconsultation, (8) online therapy, (9) virtual health care, and (10) digital health. These key terms were derived from preliminary searches, relevant Medical Subject Headings (MeSH) headings, and prior known terminology seen in policies. During the full-text review, each article was fully read and tagged with key concepts that related to any of the aforementioned keywords (1–10). Database search strings were constructed using “OR” to combine overlapping terms and “AND” to connect major concepts. These tags were grouped into categories for easier thematic analysis of the articles and eventually synthesized into overarching themes. The specific search strings are included in Appendix B. Tagging was performed by AB, with any points of uncertainty during the process resolved through discussion with JC. Of note, although primary care was not a key search term, it was part of the inclusion criteria during study selection. This decision was made because of the fact that explicitly adding “primary care” to the search string may have led to premature exclusion of articles that still aligned with the core research objective.

## STUDY SELECTION

Database searches yielded 246 references, with a final search performed on October 18, 2024.

Covidence’s (Melbourne, Australia) online screening software was used to undertake 2 screening stages. Upon removal of duplicate results, 137 articles underwent title and abstract screening (AB).

Any decision conflicts that emerged in this stage were resolved via discussion of rationale (JG, AB, JC), with 76 ultimately having met the criteria for full-text review, of which 4 were irretrievable. A full-text screening (AB and JG) was conducted for the remaining 72 studies, once again resolving any conflicts via discussion, excluding multiple studies that fit the exclusion criteria of no full-text available, wrong setting, wrong intervention (misinterpretation of “waivers”), and a wrong focus (not focused on primary care). From this careful screening, 19 articles that met all evaluation criteria were included in this review. The PRISMA flow diagram in Figure 1 details this process.

**Figure 1. A Preferred Reporting Items for Systematic Reviews and Meta-Analyses (PRISMA) Flow Diagram Detailing the 2-Stage Screening Process.**
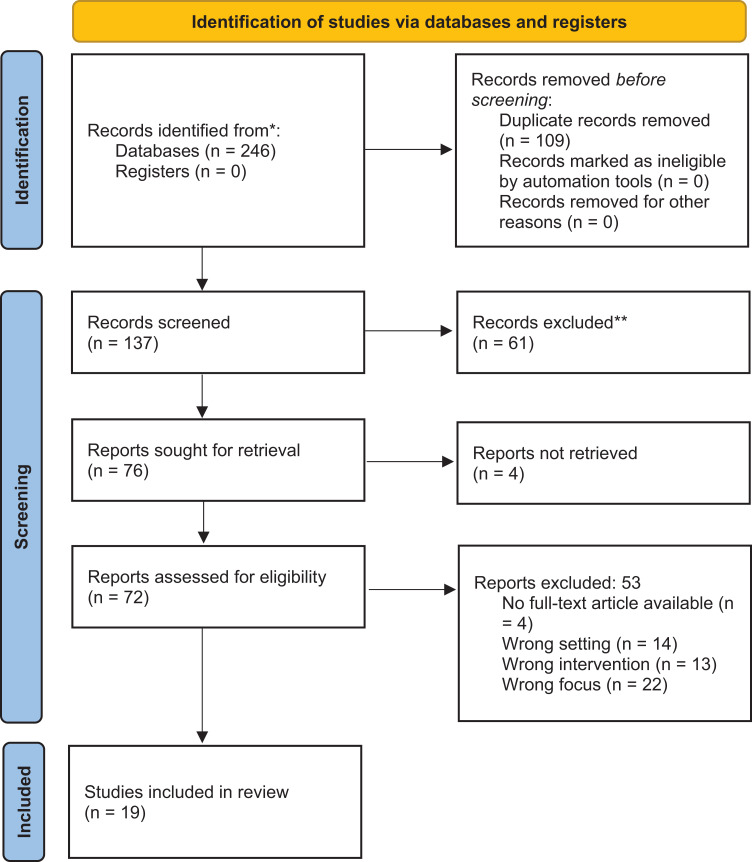
Of the 246 records initially identified through database searches, 109 duplicate entries were removed before screening. The remaining 137 records underwent title and abstract screening, of which 76 were sought for full-text review. Of these, 4 could not be retrieved. Among the 72 full-text articles reviewed, 53 were excluded for reasons that include wrong setting, intervention, or focus, as well as lack of full-text access. In total, 19 studies met all of the inclusion criteria and were included in this scoping review.

## RESULTS

### Main Findings

Of the 19 studies analyzed, retrospective studies were the main research method among a majority of sources (18; 94.7%), relying primarily on qualitative data or reports. One study supplemented the qualitative findings with a particular indexing tool, namely, the ADI—when consolidating their results.^6^ Notably, with the exception of 1 source,^6^ none of the articles relied solely on an existing index. Regarding telehealth utilization trends, a recurring pattern across the studies was that removal of the geographic restrictions provided the most significant benefit of the expanded 2020 Medicare coverages and policy waivers, particularly for disadvantaged or rural communities. Out-of-state and rural communities were among the most affected because they could not avail these waivers.^4,10^ Consequently, these same populations—particularly those receiving care across state lines—are less likely to receive both telemedicine (adjusted odds ratio [AOR], 0.90; 95% confidence interval [CI], 0.84–0.96) and in-person (AOR, 0.73; 95% CI, 0.68–0.77) visits following the waiver expiration.^11^

### Overall Timeline Impact

**Figure 2. Weekly Rate of Telemedicine Visits (per 100 Outpatient Visits) Across Area Deprivation Index (ADI) Quartiles, 2019–2021.**
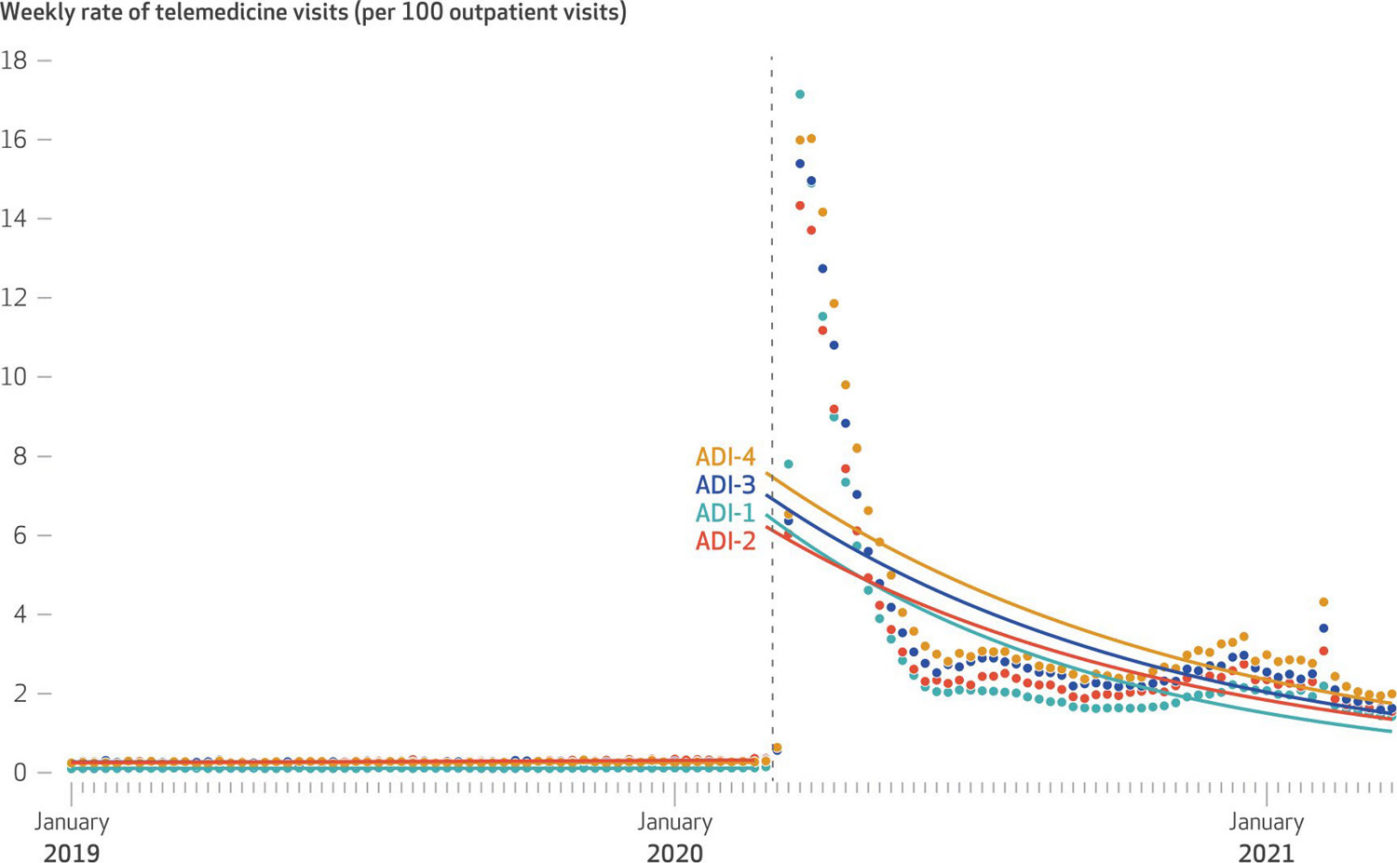
This figure displays how telemedicine use changed over time by ADI quartile. Before 2020, usage was relatively consistent across all groups. Once federal waivers were enacted (dashed vertical line), there was a steep spike in telemedicine visits across the board—with ADI-4 (least disadvantaged) communities seeing the highest immediate increase. Although rates declined over the following months, they still remained higher than prewaiver levels.

Before the waivers, telemedicine usage was considered relatively consistent overall and within each ADI quartile. After the waivers, usage increased, which correlated with higher ADI scores. This outcome is illustrated by the time series analysis in Figure 2 that shows the results before and after telemedicine waivers were initiated.^6^ In particular, in the week following waiver adoption, telemedicine use significantly increased across all groups in the study, including a 56.0-fold (95% CI, 12.3–253.7) increase in the least disadvantaged neighborhoods and a 28.9-fold (95% CI, 10.4–79.9) increase in the most disadvantaged neighborhoods (all *P* < .001), suggesting that structural access barriers (which include broadband, disability access, insurance status), not lack of interest, had previously constrained telehealth uptake.^6^ Without the waivers, patients did not simply switch their appointments from telemedicine to in-person visits; many chose to forgo care altogether.^11^ This highlights an urgent concern for health care access: Patients valued telehealth so highly that when it was no longer available, they opted to skip routine check-ins rather than face the barriers of in-person care. Without telehealth—or with restrictions reintroduced—this shift in accessibility disproportionately affected nonambulatory, financially disadvantaged, transportation-dependent, or rural populations who struggled to attend in-person visits. With >92% of the 52.7 million Medicare telehealth visits in 2020 having taken place from the patient’s home, the demand exists, but the means to sustain and expand this essential service falls short.^4^

### Key Themes

Notably, a common theme across the studies is that the focus has shifted away from promoting telehealth awareness; instead, the attention has turned to removing regulatory and implementation barriers to meet ongoing demand.^6,10,12,13^

This shift directly aligns with improvements in care logistics and quality. Koumpias et al^12^ and Hartzell et al^13^ highlighted that both patients and providers regarded telehealth as a more affordable option. Telehealth reliance has also consistently increased in geographically underserved areas. These findings reinforce that continued investment in telehealth expansion not only supports access but also allows providers—and medical facilities by extension—to conserve time and resources, ultimately enhancing quality of care and supporting long-term economic sustainability.^12,13^ In other words, an investment in telehealth—and more targeted, enhanced utilization for populations who benefit most, especially those in geographically limited, disadvantaged, and rural communities—translates into more equitable access, greater system efficiency, and an improved continuity of care. Each study in our review emphasized different variables as seen in Appendix A.

## DISCUSSION

This review sought to identify the factors, especially federal policy waivers, that influence telehealth utilization in primary care. Each study in our review emphasized different variables. Our findings indicated that one of the main impacts on telehealth utilization was in fact the Medicare telemedicine coverage waiver, which significantly increased telehealth access for previously underserved populations, especially rural communities and non–Medicaid-funded patients. Yet these waivers also exposed persistent geographic and accessibility disparities.^11–13^ As Hartzell et al noted,^13^ telehealth is still considered an underutilized novelty for certain medical organizations, providers, and particular populations around the country. Despite its widespread adoption, gaps in accessibility clearly continue to limit utilization. Before the Medicare telemedicine coverage waiver, telemedicine was mainly used in certain medical facilities and designated geographic areas.^11^ Although waivers expanded access, the demand for telehealth seems to have outpaced its availability, leading to inequities in utilization.^13^ Bose et al^6^ highlighted this concern that while the findings are promising in regard to the waiver’s intended impact coming to life, there is a further need for interventions that target telemedicine access to populations in need. Our review underscores the importance of long-term policy solutions to ensure that telehealth remains an accessible and even sustainable health care option. Of note, a common theme among the articles was that the focus has significantly shifted—from raising awareness about telehealth to removing regulatory and implementation barriers to more effectively meet the demand.

A common occurrence in the majority of the studies we reviewed was a heavy reliance on qualitative data, with the exception of 1 source relying strictly on a quantitative indexing tool (ADI) to showcase results.^6^ This observation highlights that more comprehensive quantitative tools in the context of policy changes and quantitative analyses are needed in this domain, which currently seem limited. As Snyder et al^14^ relayed, the benefit of more comprehensive outcomes data regarding factors such as the impact of telehealth on patient safety and quality of care is necessary with its uphill growth and popularity. This would allow a more tangible assessment of the waivers to be discussed at length, particularly when comparing populations and variables across distinct geographic pockets within individual states and across states more broadly.

Furthermore, the geographic limits on access to health care are more effectively addressed through telehealth. Among the studies discussed in this review, 11 heavily emphasized the specific impact of telehealth in minimizing and addressing these widespread geographic barriers—from rural to out-of-state comparisons.^4,6,10–17^ This outcome highlights the importance of telehealth as a tool for fundamental access to care—again, meeting patients where they are, rather than requiring them to bear transportation costs or navigate the logistics of reaching the nearest hospital that will offer the services they need.

Expanding on the disparities revealed by the waivers, the role of state-level policies adds another layer of complexity to the overall picture of telehealth utilization. The marked differences in health care access for out-of-state patients highlight the significance of state-specific licensure regulations and their impact on telehealth adoption. States across the country have begun recognizing the value of letting clinicians use telehealth across state lines, as shown by utilization trends during the waiver period. As of April 18, 2022, 15 states had licensure flexibilities in place to allow out-of-state clinicians to deliver care via telehealth, and several others had passed similar legislation.^10^ This ties to how utilization patterns are conditioned by state-level policies, particularly which states allow out-of-state telehealth and which do not, making it difficult to establish a national standard or draw seamless comparisons in utilization.

By the same token, although the waivers are arguably one of the most popular contributing factors to the increase in telehealth use and reliance, these are not the sole reason that patients eventually decide to accept or decline telehealth services. Although multiple studies in this review relay this result, certain studies particularly note that improving access to health care is not simply a matter of initiating a waiver but that sustainable access to telemedicine starts by alleviating overlooked factors such as technological accessibility, lack of high-speed internet—which >20% of Americans in rural areas cite^1^ as a challenge—disability accessibility, dual-eligibility statuses.^1,12,14^ A clear pattern emerged across most of the studies in this review, once again highlighting the urgent need for targeted practices and interventions to help resolve each factor.

This review highlights the persistent gaps in telehealth accessibility, which directly affect its utilization. To address these issues, strategic policy measures and further research are necessary. For instance, findings on the immediate utilization effects of telehealth waivers consistently indicate that rural populations and patients near the state borders benefit the most from relaxed regulations. Yet, paradoxically, these same groups continue to face significant barriers to telehealth access, ranging from limited digital literacy to unreliable broadband connectivity.^1,6,12,13,15,17^ These findings suggest the need for targeted, state-specific policies rather than uniform nationwide approaches, ensuring that resources and infrastructure investments address local needs. Such policies would enable more effective resource allocation to overcome barriers and support in-depth studies on patient outcomes and satisfaction.^10^

Furthermore, this review found that only 1 study used a comprehensive quantitative index to evaluate telehealth outcomes while the majority of eligible studies relied heavily on qualitative data. This observation highlights a critical gap in the literature: the need for robust, standardized quantitative measures that can holistically assess patient outcomes and satisfaction across diverse state contexts on telehealth utilization before and after waivers.

In addition, determining the cost–benefit ratio of implementing more targeted, state-specific policies might help in assessing their potential to reduce disparities. In particular, cross-state comparisons, reasons for telehealth needs, and patient preferences could be some of the missing pieces as to moving forward. In terms of the clear benefits of investing in telehealth, a 2020 *Morbidity and Mortality Weekly Report* cited telehealth as the primary driver behind increased health care access, decreased disease burden for both patients and providers, and reduced need for personal protective equipment, overburdened providers, and limited provider capacity.^18^ More focus should be on continuing the use of telehealth given its advantages of increased efficiency and overall positive impact on multiple levels in the US health care system. In fact, the use of telehealth could allow for triaged patient care, in which high-risk and acute patient cases being identified and prioritized over nonurgent cases that can be accommodated via synchronous or asynchronous telehealth.^18^ Thus, the efforts toward telehealth can only aid in the efficiency of health care and access to quality care—serving all sides of the equation. Once again, future telehealth expansion should be guided by patient-centered data, infrastructure investment, and evidence-based policy adjustment—because an increase in patient interest in telehealth is not correlative to easier access.^1,13^

### Limitations

This review has several limitations. First, although all included studies met the eligibility criteria, some tended to focus on individual state statistics rather than national, more broader trends, which may have skewed the overall interpretation of telehealth utilization patterns. Second, although the review aimed to assess primary care telehealth usage, some studies included both primary and acute care settings. Although the primary care data were the main focus of this review, this distinction was not explicitly outlined in the inclusion or exclusion criteria. In addition, conflicting findings across studies required some careful interpretation. Most sources indicated that rural populations had a greater need for telehealth support and that prewaiver telehealth was primarily used in urban settings. However, certain findings presented an alternative perspective, claiming that the odds of telemedicine use were more than 4 times higher in rural areas than in metropolitan areas in the prewaiver period, as those patients were generally the only ones eligible to use telemedicine.^6^ Such contradictions highlight the complexity and intricacies of telehealth access and suggest that geographic disparities may not be uniform across all patient populations. This outcome highlights how telehealth utilization patterns are contingent on whether individual states permit out-of-state telehealth, making it difficult to establish a consistent national standard or make direct comparisons across state lines. Moreover, the data sets used across studies varied because this review examined national telehealth utilization trends, whereas many other studies relied on regional- or state-level data, potentially limiting the generalizability of the overall findings.

## CONCLUSIONS

This scoping review examined the various factors contributing toward the disparities in telehealth utilization across the United States, with particular focus on the effects of federal waivers and the persistent barriers faced by rural populations and those living near state borders. Although policy waivers have shown immediate benefits for these groups, structural barriers continue to hinder equitable access. The evidence suggests that a one-size-fits-all policy may not be effective; instead, making waivers permanent in states where data show clear benefits could support more targeted efforts. Since the final October 2024 search, significant policy updates have occurred: Congressional and Centers for Medicare & Medicaid Services (CMS) actions, including provisions in the December 2024 American Relief Act, have extended most Medicare telehealth waivers through September 30, 2025.^19^ Further advancements include the extension of key telehealth flexibilities through December 31, 2025: continued authorization of audio-only telehealth for home-based care and the continued suspension of limits on successive inpatient visits, critical care consultations, and services in nursing facilities.^19^ Future research should focus on the more long-term health outcomes of telehealth usage, particularly in rural and underserved populations, and explore scalable alternatives to the current onesize-fits-all policy to help bridge those persistent gaps in telehealth access. Ultimately, telehealth utilization hinges on not only sustaining the momentum caused by the telehealth policy waivers but also addressing the deeper structural, geographic, and technological barriers that continue to shape patient care and access, highlighting the need for policies that are as dynamic and inclusive as the populations they are aiming to serve.

## AUTHOR CONTRIBUTIONS

AB, lead author, contributed to writing original draft of the manuscript; JG and SK, lead authors, contributed to writing, review and editing of the manuscript; JC, contributing author, methodology development and interpretation of data. All authors reviewed and approved the final version of the manuscript.

## DISCLOSURES

The authors have nothing to disclose.

## FUNDING

This study was supported by the National Center for Advancing Translational Sciences of the National Institutes of Health (RC2TR004380). The content of this article is solely the responsibility of the authors and does not necessarily represent the official views of the National Institutes of Health, nor does its mention of department or agency names imply endorsement by the US government. All authors had access to the data and a role in writing the manuscript.

## APPENDIX A

**Table:**

**Research title and authors**	**Aim of the study**	**Research methodology**	**Population and sample size**	**Main findings**	**Strengths of research/methodology**	**Weaknesses of research/methodology**	**Implications for practice**
Telehealth expansion and Medicare beneficiaries' care quality and access. Saharkhiz M; Rao T; Parker-Lue S; Borelli S; Johnson K; Cataife G	Comparing and observing the association of telehealth use with health care outcomes to determine whether telehealth waivers implemented during the COVID-19 pandemic should be made permanent.	Retrospective cohort study across 3436 hospital service areas using a population-based difference-in-difference (DID) framework.	3436 hospital service areas (including approximately 30 million Medicare beneficiaries)	High levels of telehealth use were associated with more clinician encounters, more ACS hospitalizations, and higher total health care costs.	Strong alignment with the systematic review’s research question; data from US Medicare FFS population; robust DID methodology.	Does not include postpandemic outcomes; results may not generalize outside the PHE context.	Telehealth expansion increased access but may have reduced quality (as shown by ACS hospitalization increases). Post–COVID-19 replication studies are needed to fully understand the impact of telehealth waivers.
Expiration of state licensure waivers and out-of-state telemedicine relationships. Bressman E; Werner RM; Cullen D; Ukert B; Barsky BA; Kowalski JL; Mehrotra A	Comparing out-of-state telemedicine relationships in states where licensure waivers expired vs states where waivers continued.	Cross-sectional study using Elevance Health claims data (January 2019–June 2022) with multivariate logistic regression. Models adjusted for patient demographics, geographic factors, and relationship characteristics. Subgroup regressions were conducted by specialty, preperiod visit count, distance, and licensure status.	Patients living in 3 states where waivers expired (Colorado, Maine, Wisconsin) and 5 states where waivers continued (California, Georgia, Indiana, New Hampshire, New York).	Out-of-state telemedicine visits increased, while the waivers were active. When waivers expired, patients did not switch to in-person care but instead often stopped seeing the physician altogether.	Models looked at demographic, geographic, and relationship characteristics; included subgroup analyses.	Limited sample of states with accessible data; heterogeneity between state populations; potential residual confounding.	Findings strongly support the need to reform state licensure. Continuity did not decrease for out-of-state clinicians with proper licensure. Expiration did not immediately stop out-of-state telemedicine, suggesting clinicians might not be aware of evolving rules—warranting further regulatory investigation.
Telehealth reform post**-**public health emergency: Crucial next steps. Thomas D; Garate D; Fu S; Bashir A; Moss N; Nair M	Considering policy implications and imperatives necessary in future legislation.	Retrospective review synthesizing multiple studies to create a timeline of events and derive policy implications.	NA—This is a policy-focused review article.	If restrictions return, individuals who are nonambulatory, financially disadvantaged, lack transportation, or reside in rural areas would face major barriers—making a “telehealth cliff” likely.	Important overview for contextual background; strong policy relevance for a systematic review; clear implications that can be used in conclusions/discussion.	Not a traditional empirical study; lacks sample size, statistical methods, or population data.	Telehealth already supports marginalized patients effectively but requires permanent removal of outdated restrictions. Legislative modernization is critical so telehealth can continue improving access and equity for Medicare patients.
Association of licensure and relationship requirement waivers with out-of-state tele-mental health care, 2019–2021. Koumpias AM; Fleming O; Lin LA	Observing telehealth policy variation to estimate the association of in-state licensure waivers and pre-existing patient-physician relationship requirement waivers.	Retrospective study using change health care claims data (January 2019–December 2021). Analyzed number of monthly mental health visits via DID models and event-study falsification tests.	2 037 977 commercially insured patients across 3 Midwestern metro areas (Chicago, Davenport, St. Louis). Included only patients under 65 with ≥1 outpatient mental health visit in 2019 and a laboratory-confirmed COVID-19 diagnosis.	Licensure waivers during the PHE were associated with an ∼16-fold increase in out-of-state telemental health visits. Removing regulatory barriers effectively expanded access but reduced the relative share of out-of-state telemental health within all telehealth mental health visits.	Direct observation of telehealth utilization; strong quantitative analysis; rigorous falsification tests strengthen validity; limited to only 3 MSAs; focuses solely on tele–mental health rather than broader telehealth usage.	Highlights the importance of maintaining licensure flexibility, especially for patients near state borders. Useful for shaping policy around waiver expiration and mental health access.	The findings contribute to the ongoing policy discourse on waiver expiration by highlighting the relatively greater importance of out-of-state tele–mental health care for patients residing near state borders.
Medicare and telehealth: The impact of COVID-19 pandemic. Hamadi HY; Zhao M; Haley DR; Dunn A; Paryani S; Spaulding A	Looking into the impact of changes in Medicare policy (waivers during the PHE) on the proliferation and utilization of telehealth services during the COVID-19 pandemic.	Retrospective analysis. Examined utilization patterns using 5 dependent variables (yes/no questions) and 8 sociodemographic variables. Descriptive analyses were performed.	9686 Medicare beneficiaries.	No main findings, but demographic factors strongly influenced telehealth availability and use. Age consistently predicted both access to and utilization of telehealth.	Considers a broad variety of demographic trends; offers important insight into Medicare beneficiary telehealth use during the COVID-19 pandemic; relevant to understanding waiver impact.	Relies on self-reported survey data; low minority representation; findings not generalizable to all Medicare beneficiaries; cannot determine reason for visits or diagnoses.	With the end of the PHE, policymakers must decide which telehealth flexibilities to retain, modernize reimbursement frameworks, and address broadband disparities—especially in rural and underserved communities.
Remote patient triage: Shifting toward safer telehealth practice. Kobeissi MM; Ruppert SD	Discussing current issues affecting telehealth and proposing recommendations for safer virtual care, especially regarding remote patient acuity and triage.	Retrospective review of prior appointment data and observed best practices.	NA	Highlights that audio-only visits were not considered “telemedicine” prepandemic and emphasizes a 2-step triage approach to prevent underestimating illness severity and to ensure patient safety.	Expansive discussion across telehealth specialty settings; clearly outlines factors influencing appropriateness of telehealth visits; directly ties into utilization/safety themes.	No specific population or data set; lacks empirical testing of the triage model.	Standardizing triage processes is essential for safe and efficient telehealth utilization; moving from reactive to deliberate telehealth will improve quality and protect patients.
Telemedicine in a primary care clinic in Fairbanks, Alaska: Not a magic bullet for providing treatment during COVID-19. Hartzell SY	Looking into the barriers faced by 2 family practice doctors in Fairbanks, Alaska, when implementing telemedicine during the COVID-19 pandemic.	Qualitative analysis based on discussions with 2 family practice doctors during the COVID-19 pandemic.	Two family practice doctors in Fairbanks, Alaska.	Telemedicine use increased due to the COVID-19 pandemic, offering benefits such as limiting exposure and increasing access to specialty care.	Provides real-world insights into rural primary care challenges; demonstrates lived provider experiences.	Extremely small sample size; qualitative only; not representative; lacks quantitative outcomes.	To improve telemedicine adoption in underserved areas, systems need provider incentives, insurance waivers for necessary equipment, and patient education on when/how to access virtual care.
A year after implementation of the telehealth waiver: being offered and utilizing video specific telehealth among dual eligible Medicare recipients during the COVID-19 pandemic. Choi J; Kim G; Choi S; Chang JE	Examining factors associated with telehealth availability and usage among Medicare and dual-eligible patients 1 year after Medicare’s temporary telehealth waiver.	Cross-sectional phone survey using the Winter 2021 MCBS (Medicare Current Beneficiary Survey) data set.	Population sample of 10 586 Medicare recipients.	Those who were dual-eligible and had severe chronic conditions were more likely to have telehealth visits, whereas people in metropolitan areas were less likely to have been offered telehealth. The federal waivers managed to effectively maintain access for vulnerable Medicare recipients.	Large, nationally representative sample; strong demographic variation.	The time frame is only pertinent to the COVID-19 pandemic period, which might not give generalizable results.	Telehealth waivers successfully supported care continuity for high-need groups. Policymakers should consider maintaining flexibilities for vulnerable populations.
Medicare beneficiaries in disadvantaged neighborhoods increased telemedicine use during the COVID-19 pandemic. Bose S; Dun C; Zhang GQ; Walsh C; Makary MA; Hicks CW	Looking into the ADI to observe socioeconomic disadvantage and its association with telemedicine use over time.	Retrospective cohort study using Medicare fee-for-service claims data (January 2019–March 2021).	30,588,891 Medicare beneficiaries with at least 1 outpatient visit claim to Medicare.	Before the waivers, the weekly rate of telemedicine was relatively stable, yet after the waiver was announced, an increase in telehealth usage was observed.	Very large sample; strong pre- or postwaiver comparison; directly addresses utilization patterns.	Focused only on Medicare beneficiaries; does not include specific applications of telemedicine usage; the generalizability of results is limited outside of Medicare.	Further research needs to be done to explore what drives telemedicine access. Ensuring timely access during and beyond COVID-19 is essential for all socioeconomic groups.
Unpacking a telemedical takeover: recommendations for improving the sustainability and usage of telemedicine post-COVID-19. Kaundinya T; Agrawal R	Reviewing the overall state of telehealth, before, during, and after the COVID-19 pandemic, including utilization, coverage, privacy, and long-term sustainability needs.	Cross-sectional study that used national survey data from JD Power briefly, but no consistent use of quantitative data sets across the article.	NA	Telemedicine adoption and utilization have increased over the course of the COVID-19 pandemic. For it to remain a long-term component of the US health care system, major updates in funding models, licensure rules, coverage criteria, and telemedicine boundaries are necessary.	Provides strong policy reasoning and a comprehensive view of the system changes needed to sustain telemedicine; greatly helpful for framing policy in the future.	Limited in its quantitative evidence; there is a lack of defined population statistics to back up the data; relies heavily on narrative review.	Targeted investment in telemedicine could expand patient choice, reduce provider burnout, and support telemedicine’s long-term stability.
Trends in the use of telehealth during the emergence of the COVID-19 pandemic—United States, January 2020. Koonin LM; Hoots B; Tsang CA; Leroy Z; Farris K; Jolly T; Antall P; McCabe B; Zelis CBR; Tong I; Harris AM	Assessing changes in telehealth usage and utilization patterns during the early stages of the COVID-19 pandemic.	Analysis of data from 4 large national telehealth providers comparing January to March 2020 to the same period in 2019.	Data from 4 large US telehealth providers (broad national sample).	Telehealth visits increased by 50% during the first quarter of 2020 vs 2019, with a particular 154% increase in the last week of March 2020. COVID-19–related encounters increased from 5.5% to 16.2%.	Used data from multiple large telehealth providers and thus provided a broader perspective on national trends.	Data were limited to sampled providers; symptoms for identifying COVID-19 were nonspecific, causing misclassification risks.	The significant increase in telehealth usage suggests that the policy changes and public health guidance during the pandemic effectively promoted virtual health care/telehealth as a whole. This analysis helped determine patterns of health care delivery modalities that are useful for planners and providers.
Telehealth as an effective care delivery method during the COVID-19 pandemic for the Rhode Island behavioral health population. Flowers D; Goodspeed E; Daly M	Evaluating the effectiveness of telehealth in providing behavioral health services during the COVID-19 pandemic in Rhode Island.	Retrospective analysis of telehealth utilization and patient engagement data across behavioral health providers.	Rhode Island behavioral health patients.	Telehealth increased overall access and engagement. The average total visits rose from 36.7 prewaiver to 38.4 postwaiver. However, digital literacy and internet access barriers persisted for certain populations.	Highlighted real-world application of telehealth in a specialized population, with demonstrated improvements in service utilization.	Narrow geographic scope; reliant on self-report variables; lacks long-term outcomes and non-Medicaid population data.	Telehealth is a viable mode of behavioral health care, but successful telemedicine requires addressing digital literacy gaps and expanding infrastructure support.
Erratum: Trends in the use of telehealth during the emergence of the COVID-19 pandemic—United States, January-March 2020 (*Morbidity and Mortality Weekly Report* 69:43 (1595) DOI: 10.15585/mmwr.mm6943a3)	Providing corrected data set and expanded details on early pandemic telehealth use for monitoring, planning, and improving health outcomes during the COVID-19 pandemic.	Cross-sectional analysis of 4 national telehealth provider data sets that examined demographics, geography, encounter type, and disposition.	National telehealth provider data.	Telehealth served an important role in the pandemic response. Waivers and public guidance likely increased adoption and overall telehealth encounters varied by age, sex, and region.	Provided a detailed demographic and geographic breakdown; helps refine the understanding of early pandemic telehealth patterns.	Provider sample not fully representative; early COVID-19 symptoms overlapped with other conditions, causing identification challenges.	Expanded telehealth access and supportive policies may continue benefiting patients and providers beyond the pandemic.
The impact of COVID-19 on financing of psychiatric services. Miller LH; Parks J; Yowell R	Examining the financial effects of the COVID-19 pandemic on behavioral health service delivery, including governmental interventions and the shift to virtual care.	Review of existing data and literature on psychiatric service financing during the pandemic.	NA	The COVID-19 pandemic significantly disrupted psychiatric service financing. Governmental agencies provided financial support, but measures did not fully offset the pandemic impacts.	Comprehensive analysis of existing data provides a broad understanding of the pandemic's impact on psychiatric services.	Relies on secondary data; dependent on quality and completeness of existing studies.	Highlights the need for effective leadership, communication, and coordination to strengthen psychiatric service financing and pandemic preparedness.
Interstate telehealth use by Medicare beneficiaries before and after COVID-19 licensure waivers, 2017-20. Andino JJ; Zhu Z; Surapaneni M; Dunn RL; Ellimoottil C	Analyzing trends in interstate telehealth use among Medicare beneficiaries before and after the COVID-19 pandemic licensure waivers and looking at how these waivers affected access.	Retrospective analysis of Medicare fee-for-service (FFS) outpatient evaluation and management claims between 2017 and 2020.	Medicare FFS beneficiaries who used outpatient evaluation and management services (exact sample size was not stated explicitly).	Telehealth usage increased 63-fold in 2020, but the proportion of interstate telehealth visits remained relatively stable. Overall, rural beneficiaries used more out-of-state telehealth than did the urban ones. Significant variation existed across states.	Uses national Medicare data over multiple years; provides insight into policy impacts and state-level variation.	Sample size was not explicitly stated; does not control for all technological or preference-based barriers; potential unmeasured confounders.	Policymakers should consider state-specific needs when deciding whether to maintain licensure flexibilities. Waivers may improve access, especially for rural patients and states dependent on out-of-state clinicians.
Telehealth billing for nurse practitioners during COVID-19: Policy updates. Snyder EF; Kerns L	Providing an overview of essential telehealth policies, legislative updates, and resources to help nurse practitioners (NP) on contemporary telehealth billing regulations during the COVID-19 pandemic.	Retrospective policy overview that reviewed federal and state telehealth legislative changes during the pandemic.	NA	Telehealth became widely adopted to maintain access during the pandemic. Multiple temporary waivers and billing exceptions were introduced in 2020. The article includes resources and websites for NPs on accurate and legal telehealth billing.	Timely and practical; provided actionable billing guidance; summarized rapid regulatory changes clearly; highly relevant for NP practice.	There was no empirical data; relies heavily on evolving policies that may quickly become outdated.	NPs need to stay current with policy updates to ensure accurate telehealth billing and compliance. Continued education is essential as telehealth regulations evolve beyond the pandemic.

Abbreviations: ACS, Ambulatory Care–Sensitive; DID, Difference-in-Differences; FFS, Fee-for-Service; JD, J.D. Power (market research and analytics firm); MSA, Metropolitan Statistical Area; PHE, Public Health Emergency.

## Appendix B

In CINAHL, the following search string was used: (MH “Digital Health+”) OR MH “Telehealth+”) OR (“digital health” OR “digital healthcare” OR “virtual care” OR “virtual healthcare” OR “telemedicine” OR “tele-medicine” OR “telehealth” OR “tele-health” OR “Digital Health” OR “Telemedicine”).

In Scopus, the following search string was used. TITLE-ABS (“digital health” OR “digital healthcare” OR “virtual care” OR “virtual healthcare” OR “telemedicine” OR “tele-medicine” OR “telehealth” OR “tele-health” OR “Digital Health” OR “Telemedicine”) AND TITLE-ABS (“waiver” OR “waivers”).

In PubMed, the terms used were “telehealth” OR “tele-health” OR “digital health” OR “digital healthcare” OR “virtual care” OR “virtual healthcare” OR “telemedicine” OR “tele-medicine” OR “Digital Health” OR “Telemedicine”).

In PsycInfo, the terms used in the search string were (DE “Telemedicine” OR DE “Online Therapy” OR DE “Teleconferencing” OR DE “Teleconsultation” OR DE “Telepsychiatry” OR DE “Telepsychology” OR DE “Telerehabilitation”) OR (DE “Digital Interventions” OR DE “Mobile Health” OR DE “Mobile Health Applications” OR DE “Wearable Devices”) OR (“digital health” OR “digital healthcare” OR “virtual care” OR “virtual healthcare” OR “telemedicine” OR “tele-medicine” OR “telehealth” OR “tele-health” OR “Digital Health” OR “Telemedicine”) AND → “waiver” OR “waivers”.
